# A novel approach identifies the first transcriptome networks in bats: a new genetic model for vocal communication

**DOI:** 10.1186/s12864-015-2068-1

**Published:** 2015-10-22

**Authors:** Pedro Rodenas-Cuadrado, Xiaowei Sylvia Chen, Lutz Wiegrebe, Uwe Firzlaff, Sonja C. Vernes

**Affiliations:** Max Planck Institute for Psycholinguistics, Wundtlaan 1, Nijmegen, 6525 XD The Netherlands; Ludwig-Maximilians-Universität, Division of Neurobiology, Department Biology II, Großhaderner Straße 2, Planegg-Martinsried Munich, D-82152 Germany; Lehrstuhl für Zoologie, TU München, Liesel-Beckmann-Str. 4, Freising-Weihenstephan Munich, 85350 Germany; Donders Centre for Cognitive Neuroimaging, Kapittelweg 29, Nijmegen, 6525 EN The Netherlands

**Keywords:** Bat, Periaqueductal gray, Vocal communication, Glutamate signaling, Co-expression network analysis, WGCNA, MCLUST

## Abstract

**Background:**

Bats are able to employ an astonishingly complex vocal repertoire for navigating their environment and conveying social information. A handful of species also show evidence for vocal learning, an extremely rare ability shared only with humans and few other animals. However, despite their potential for the study of vocal communication, bats remain severely understudied at a molecular level. To address this fundamental gap we performed the first transcriptome profiling and genetic interrogation of molecular networks in the brain of a highly vocal bat species, *Phyllostomus discolor*.

**Results:**

Gene network analysis typically needs large sample sizes for correct clustering, this can be prohibitive where samples are limited, such as in this study. To overcome this, we developed a novel bioinformatics methodology for identifying robust co-expression gene networks using few samples (N=6). Using this approach, we identified tissue-specific functional gene networks from the bat PAG, a brain region fundamental for mammalian vocalisation. The most highly connected network identified represented a cluster of genes involved in glutamatergic synaptic transmission. Glutamatergic receptors play a significant role in vocalisation from the PAG, suggesting that this gene network may be mechanistically important for vocal-motor control in mammals.

**Conclusion:**

We have developed an innovative approach to cluster co-expressing gene networks and show that it is highly effective in detecting robust functional gene networks with limited sample sizes. Moreover, this work represents the first gene network analysis performed in a bat brain and establishes bats as a novel, tractable model system for understanding the genetics of vocal mammalian communication.

**Electronic supplementary material:**

The online version of this article (doi:10.1186/s12864-015-2068-1) contains supplementary material, which is available to authorized users.

## Background

Bats are a remarkable, yet under-utilized model system in which to interrogate genetic mechanisms underlying mammalian vocal communication. Echolocating bats rely heavily on vocalisation to navigate, and different species utilize learned and/or innate communication calls to mediate complex social interactions [1–3]. In addition, increasing evidence suggests that social information (e.g. identity, sex, age) can also be encoded within echolocation calls [1]. Historically, studies in bats have largely focused on echolocation and as a result, much is known regarding production, auditory perception and sensorimotor integration of these calls at a psychophysical and neurological level [4–7].

More recently, investigations across different species have revealed astonishing complexity in the communication calls used by bats including vocal dialects, group foraging calls, distress calls, courtship and territorial songs [8–12]. In a handful of species, evidence for learning of social vocalisations has also been shown. For instance, pup isolation calls in *Phyllostomus discolor*, group signature calls in *Phyllostomus hastatus*, territorial songs in *Saccopteryx bilineata* and social calls in *Rousettus aegyptiacus* are acquired via vocal learning [3, 13–15]. The presence of this extremely rare ability, shared with humans and only a handful of other species (some birds, elephants, pinnipeds and cetaceans), recommends bats as a model not only for vocal communication, but also for the evolution and development of spoken language [15, 16].

However, despite their obvious appeal for the study of vocal communication and the fact that bats make up the second largest mammalian order they remain severely understudied, particularly at a molecular level. This is partly attributed to the difficulty in maintaining bats in captivity, their low offspring generation (often one per year) and importantly, the lack of genomic information currently available for these species. To address this fundamental gap we have performed the first transcriptome profiling of a highly vocal bat species; *Phyllostomus discolor* (*P. discolor*). Furthermore, to understand not only the gene expression patterns, but the functional networks in which these genes act we also performed the first genetic interrogation of molecular networks in the bat brain.

We chose co-expression network analysis to uncover functional gene networks, as this approach has been shown to be effective for revealing molecular networks underlying complex disorders in human tissue samples [17–20]. Several clustering techniques have been previously described for identifying closely interconnected gene expression networks from large scale expression data. These include K-means clustering [21], hierarchical clustering [22], model-based clustering (MCLUST) [23], self-organizing-maps [24], and weighted gene co-expression analysis (WGCNA) [25] among others. The performance of these methods has been extensively discussed and WGCNA and MCLUST are thought to be two of the best performing clustering algorithms available [26–28]. WGCNA is a topological-similarity based hierarchical clustering method and in recent years it has been widely used for analysing microarray and transcriptome data [19, 29]. MCLUST is based on parameter estimation via the expectation maximisation algorithm for normal mixture models [23] and has been applied to analyse both transcriptome and MRI data [26, 30]. However robust gene clustering has been estimated to require a minimum of 15 samples and thus standard co-expression gene network analysis can be prohibitive where samples are limited or difficult to obtain, such as in this study.

To address this issue, we developed a novel approach for gene network analysis. This method consists of an initial generation of gene networks using WGCNA and MCLUST followed by an iterative re-clustering procedure which sequentially refines gene networks to reduce instability resulting from using few samples. To further increase robustness, the networks produced by each method are then compared to generate consensus modules common to both techniques. Combining different clustering algorithms has been shown to provide more power and robustness in identifying stable clusters [31, 32] and should overcome inherent biases caused by individual computational methods. Therefore iterative re-clustering coupled to cross-comparison between clustering algorithms should provide an effective methodology for identifying robust biological gene networks, even when sample size is limited.

Given the great promise of bats for studies of vocal communication, we chose to apply our novel methodology for investigating a brain region fundamental to vocalisation in mammals; the periaqueductal gray (PAG) [33, 34]. The PAG is a midbrain region characterized by a high degree of bidirectional connections to many neuronal substrates including the thalamus, hypothalamus, inferior/superior colliculus, brainstem, amygdala and several cortical regions. As such, the PAG is thought to play a pivotal role as a processing and relay station, where signals received from different brain regions are gathered and broadcast to evoke cohesive responses. A key function of the PAG that is conserved across mammals is its involvement in the initiation of vocalisation. In humans, PAG lesions have been shown to cause muteness [35] and electrical/chemical stimulation of the PAG have been shown to elicit natural-sounding vocalisations in a range of species such as cats [36], squirrel monkeys [37] and rats [38]. In bats (including the model utilized herein; *P. discolor*), stimulation of the PAG induces natural sounding echolocation and communication calls [39, 40]. Together, these data provide a strong argument for functional conservation of the role of the PAG in vocalisation.

In this study, we performed RNA sequencing followed by *de novo* transcriptome assembly in brain tissue from six bats (*P. discolor*) that had been actively vocalizing. Initial analyses using standard WGCNA and MCLUST approaches in this small sample size produced unstable gene networks. However, we were able to generate robust, tissue-specific gene networks when applying our novel methodology. The most highly connected of the networks identified in our study was found in the PAG and represented a cluster of genes involved in glutamatergic synaptic transmission. Glutamatergic receptors are known to play an essential role in vocalisations elicited from the PAG [41, 42], suggesting that the gene network uncovered here is mechanistically important for vocal-motor control in mammals. These findings show that our novel gene clustering approach can reveal robust biologically relevant gene co-expression networks from small sample sizes. Moreover, this work reports the first gene network analysis performed in a bat brain and establishes *P. discolor* as a novel, tractable model system for understanding the genetics of vocal communication.

## Results

### Transcriptome assembly and differential expression in the bat brain

**Fig. 1:**
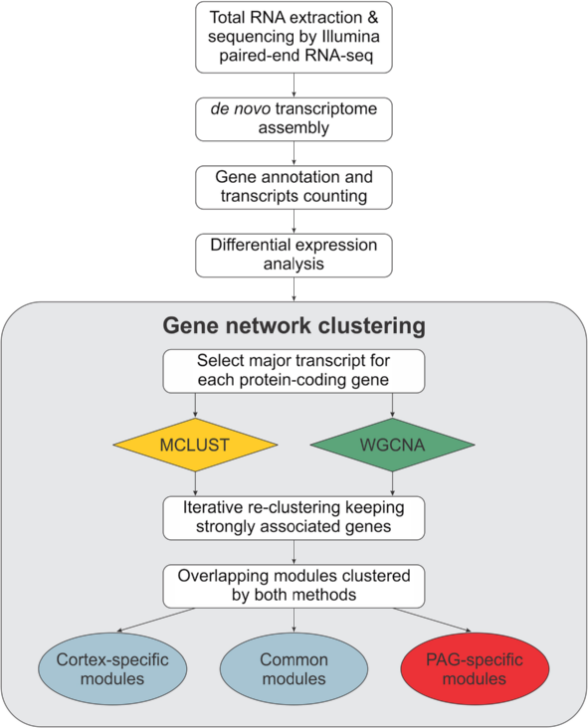
Strategy for identifying biologically relevant modules via network clustering analysis. Total RNA was extracted from the PAG and cortex of 6 adult bats. Sequencing was performed via Illumina paired-end RNA-Seq, followed by *de novo* transcriptome assembly. Following gene annotation and transcript quantification, it was possible to identify genes that were differentially expressed between the cortex and PAG. The major isoform of each protein coding gene was then selected to perform cluster analysis. Co-expressed genes were identified using two contrasting analysis models simultaneously on the same dataset; MCLUST and WGCNA. For both methods, to ensure high stringency, iterative re-clustering was performed to remove genes that were not strongly associated with the co-expressed modules. For each brain region, the modules identified via the contrasting techniques were compared in a pairwise fashion, and the most highly conserved networks were identified. Furthermore, these highly conserved networks were then compared across regions. This allowed us to identify a set of high confidence co-expressed transcriptional networks related to the PAG

To assess gene expression and co-expression networks from the bat brain, we obtained RNA from fresh tissue punches of six actively vocalising adult bats (Additional file 1: Figure S1). Tissue punches were collected from the PAG and dorsal region of the cortex at the same tissue depth, corresponding to the visual cortex (Additional file 1: Figure S1). Genome wide transcript levels were assessed via Illumina paired-end RNA sequencing (RNA-Seq). The full genome of *P. discolor* has not yet been sequenced, therefore *de novo* transcriptome assembly was performed before gene annotation and expression analysis (See Fig. 1 for work flow). Reads from seven samples (5 PAG and 2 cortex) were used for initial assembly, yielding 152,576 transcripts, 62,364 of which could be matched to protein coding genes in the Uniprot database, corresponding to 14,226 unique genes after collapsing isoforms. A small number of the remaining transcripts were identified as known non-coding RNAs, including 51 precursor microRNAs, 122 small nuclear/nucleolar RNAs and 42 cis-regulatory RNAs. The remaining transcripts could not be annotated to known genes. However given that the *P. discolor* genome has not been sequenced, these unknown transcripts may represent protein coding genes or non-coding RNAs that are poorly conserved with other species.

We then determined the abundance of all the transcripts in 12 samples (6 PAG and 6 cortex). 14,092 major isoforms (defined as the most abundant isoform) were identified in the PAG and 13,991 in the cortex. 12,221 of these isoforms were expressed in both brain regions. To test if PAG and cortex have different overall expression patterns, we first performed hierarchical clustering using either all initially assembled transcripts or the major isoform for each protein coding gene (Additional file 2: Figure S2). Both analyses separated the cortex and PAG into two clearly defined groups, indicating a unique signature of expression for each brain region. We then looked for genes differentially expressed between the cortex and PAG and found 5,264 genes after false discovery rate (FDR) correction (1 %, i.e. <0.01), (Additional file 3: Table S1). Gene ontology analysis of the top 500 overexpressed genes for each tissue was successful at identifying global functional differences, with the PAG showing enrichment in cilia related functions and the cortex in functions related to synaptic transmission and voltage gated ion channel activity (Additional file 4: Table S2). However, in order to identify more comprehensive within-tissue gene relationships, we performed co-expression clustering analysis.

### Transcriptional networks in the bat brain

In contrast to differential expression analysis where transcript abundance is averaged across samples and compared between tissues, gene network construction is based on comparison of transcript abundance across individual samples within tissues. Genes that share a covariant expression pattern across samples are then clustered together into functional subgroups. We constructed gene networks based on the co-expression patterns of the major isoform of each gene across 6 samples for either the PAG or cortex. To identify tightly co-regulated networks that would reflect transcriptional organisation, we generated gene clusters from the PAG and cortex using two contrasting methods in parallel; WGCNA and MCLUST.

These methods generally employ much larger samples sizes (N > 12) [19, 43, 44], so we hypothesised that the standard clustering approach would not have enough power to identify robust gene networks. To test this, we removed genes with the weakest cluster identity from each cluster, expecting that stable clusters should be unaffected by this. However, gene removal led to a significant rearrangement of gene-to-cluster assignment with both clustering methods, suggesting that the networks produced were not stable as small differences in the starting gene set could affect cluster membership. This suggested a substantial effect caused by uncertainty of gene-cluster identity, resulting in significant changes in how connections between genes are formed in the clusters, also called network topology. This uncertainty was likely attributed to a larger proportion of outlier genes in the data set due to the small sample size. Thus removing these outliers, that are classified as being weakly connected to clusters should improve cluster stability. In WGCNA, cluster assignment is determined by the “module membership” parameter whereas for MCLUST each gene is assigned to a cluster with an uncertainty parameter. To retain only the most highly connected genes within our network we applied an iterative re-clustering procedure that filtered out genes failing a selection threshold of module membership in WGCNA or clustering uncertainty in MCLUST. This would increase the overall identity match in the clusters, reducing the chance of uncertain gene-to-cluster assignment, and hence make clusters more distinct from each other.

**Fig. 2:**
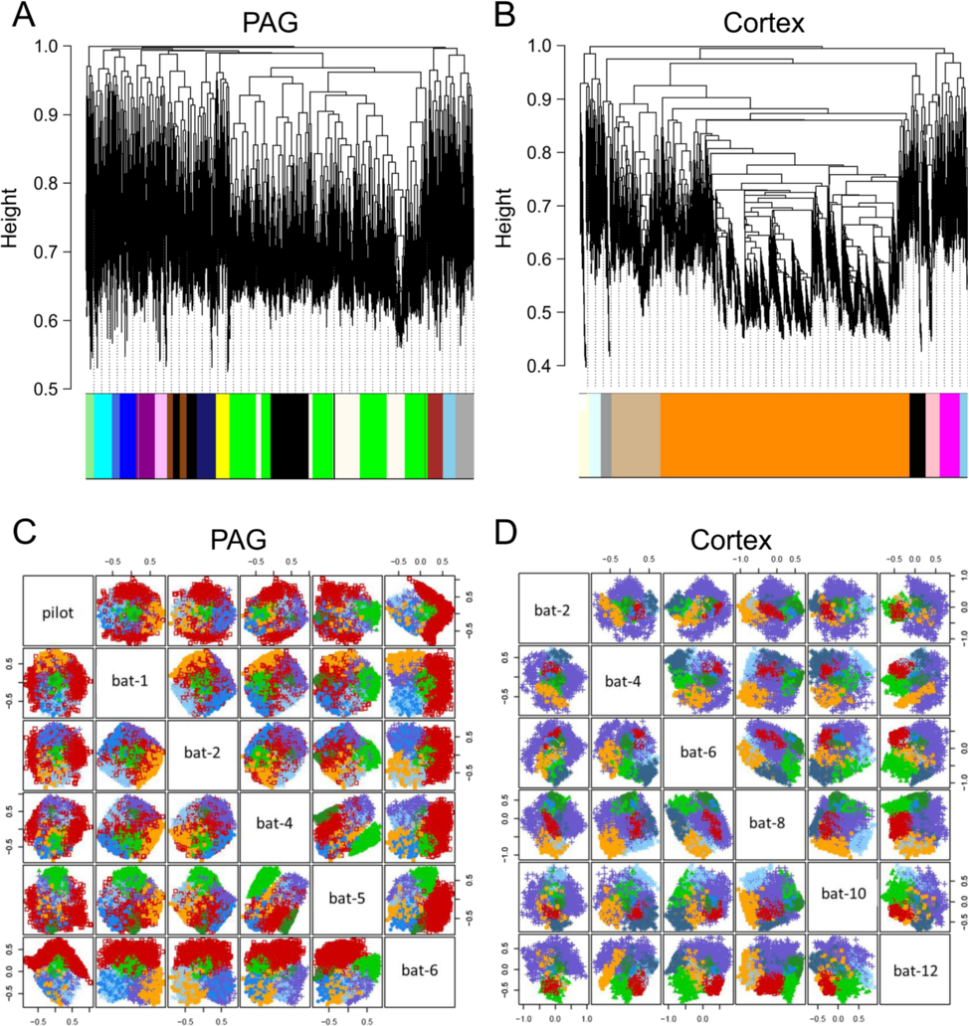
Construction of gene co-expression modules via contrasting clustering techniques. (**a-b**) WGCNA analysis generates dendrograms illustrating hierarchical clustering and modular organisation of the expressed transcripts in (a) the PAG and (b) the cortex. Genes are represented by vertical lines (leaves) on the x-axis grouped into branches, based on topological overlap (TO). Network distance is given on the y-axis (1-TO), and thus the closer the values are to 0, the more closely related the genes. Cluster colour below denotes the separation of leaves into discrete modules. In the PAG 16 modules were identified (part a), and 9 modules were identified in the cortex (part b). Consistent with the high degree of differential expression between these two tissues, the dendrogram and module structure varied greatly across the two tissues. (**c-d**) Construction of gene co-expression modules via MCLUST. Two dimensional visual representation of the six dimensional construction of the modular organisation of the PAG and cortex. Modules are displayed as groupings of coloured dots, squares or crosses. In the PAG 7 modules were identified (part c), and 8 modules were found in the cortex (part d)

The iterative re-clustering procedure started with the major isoforms. After initial clustering, genes with module membership less than 0.8 using WGCNA or genes with uncertainty greater than 0.01 using MCLUST were removed from the total gene list. These thresholds were determined to reduce the number of genes that could be classified in more than one cluster. After removal of uncertain genes, clustering was performed again with the shortened gene list and the process repeated. Module preservation was determined after each round of clustering by comparing the clusters of the current round to the previous round. The average pairwise best-matching percentages of the current modules to the previous were obtained at each round of re-clustering, as shown in Additional file 5: Figure S3. Our test showed a clear improvement in cluster stability using iterative re-clustering for both WGCNA and MCLUST (Additional file 6: Figure S4). More robust clusters were produced by the iterative re-clustering procedure as shown by an increase in average pairwise identity and a decrease in variability among clusters at each round (Additional file 5: Figure S3). The re-clustering procedure ended when all genes passed the cut-off thresholds and module preservation reached the highest point, where increasing the number of iterations could not improve the stability of the clusters. Using this method we identified 16 modules in the PAG with WGCNA and 7 modules with MCLUST (Fig. 2a & c). In the cortex, equivalent analysis identified 9 modules with WGCNA and 8 modules with MCLUST (Fig. 2b & d).

**Fig. 3:**
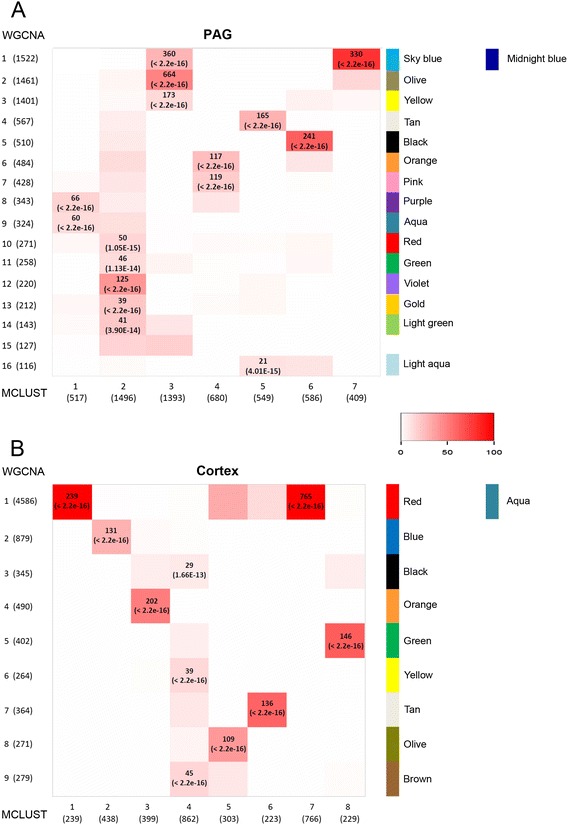
Conserved modules were identified by both the MCLUST and WGCNA methods. **a** Pairwise comparisons were made of the 16 WGCNA and 7 MCLUST modules identified in the PAG. 17 conserved modules were identified. All but one displayed a highly significant degree of overlap (*p* < 0.001). **b** Pairwise comparisons were made of the 9 WGCNA and 8 MCLUST modules identified in the cortex. 10 conserved modules were identified. All conserved modules displayed a highly significant degree of overlap (*p* < 0.001). The shading in the tables illustrates the percentage overlap as per the inset key. Significance of the most highly conserved modules for each pairwise comparison is given alongside the number of overlapping genes per conserved module. For simplicity we now assigned colour names to the conserved modules, which are given to the right of each corresponding row where the conserved module appears

Robust and biologically relevant co-expression networks should be identified by both clustering methods. Hence, to determine network reproducibility, we performed a pairwise analysis and determined the overlap between the modules produced by the two contrasting methods. For each WGCNA module we identified the most closely matched MCLUST module, and vice versa. Modules that displayed a highly significant degree of overlap (p < 0.001) were considered to be ‘consensus’ modules. From this we identified 16 consensus modules in the PAG (Fig. 3a) and 10 consensus modules in the cortex (Fig. 3b). These modules consisted of the common overlapping genes identified by both WGCNA and MCLUST. For clarity we renamed these consensus modules with colour names, as shown in Fig. 3 and unless otherwise stated, all future discussion of ‘modules’ refers to these consensus modules. Details of all modules identified in the PAG and cortex can be found in Additional file 7: Table S3 and Additional file 8: Table S4.

### Tissue specificity of consensus networks

**Fig. 4:**
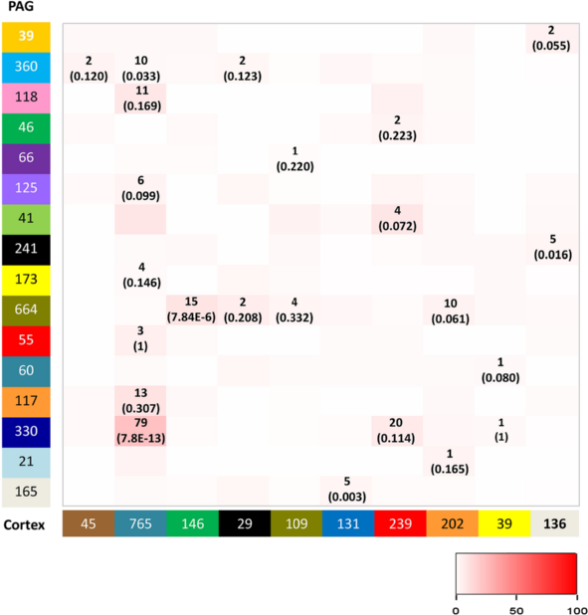
Conserved modules are not shared between the PAG and the cortex. Pairwise comparisons were made of the 16 conserved PAG and 10 conserved cortex modules from Fig. 3. The shading in the table illustrates the percentage overlap as per the inset key. Significance was calculated for the most highly conserved modules for each pairwise comparison and is given alongside the number of overlapping genes per module. Only two modules showed a significant amount of overlap (*p* < 0.001); the midnight blue PAG with aqua cortex, and the olive PAG with green cortex. However the percentage overlap was very low for both these networks (2 and 10 % respectively), suggesting that the modules identified were specific for the different tissues interrogated

**Fig. 5:**
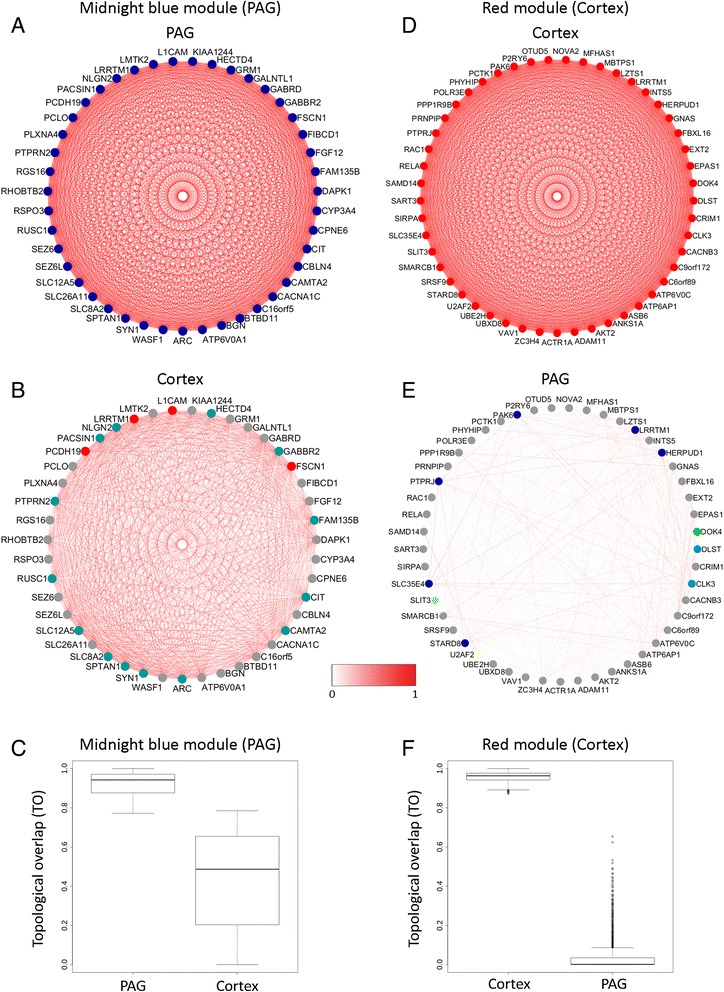
Gene connectivity is not preserved across tissues. The top 50 connected genes from the most robust modules in PAG and cortex were selected to compare how connectivity across the tissues was preserved. Connectivity was determined by the “kWithin” parameter in WGCNA, measuring the intra-modular connections weighted by topological similarities. Not all of these genes were commonly expressed in both tissues so only 43 genes from the top PAG module (midnight blue) and 49 genes from the top cortex module (red) could be compared. The gene colour represents the module they belong to and the colour intensity of gene connections indicates topological overlap (TO), also represented as “edge weight”, which is normalized between 0 and 1 for cross module comparison. The connections amongst genes for the midnight blue PAG module were not well conserved in the cortex (**a-b**). The connections between genes for the red cortex module were also poorly conserved in the PAG (**d-e**). The low preservation was also clearly illustrated when comparing the overall TO differences between the tissues for the PAG midnight blue module (**c**) or the red cortex module (**f**)

The consensus modules in PAG and cortex were identified in parallel using the same procedures. To verify transcriptional networks that were specific to the PAG or cortex, and not a general feature of neuronal tissue, we compared the PAG consensus networks with those built from the cortex. In contrast to the highly significant overlap produced by different clustering techniques within the same tissue, pairwise comparisons between PAG and cortex networks produced very little overlap (Fig. 4). To further demonstrate this, we took the most highly connected module for each tissue. For the PAG this was the ‘midnight blue’ module (Fig. 5a) and for the cortex it was the ‘red’ module (Fig. 5d). We then compared how the connectivity between top 50 connected genes for each module was conserved across tissues. In both cases, genes that were highly connected in the PAG were poorly connected in the cortex (Fig. 5a-b) and vice versa (Fig. 5d-e), indicating that the connections between genes were not well maintained across tissues. This low preservation across tissues was also clearly illustrated when comparing the overall TO differences for each module between the tissues (Fig. 5c, f). As the majority of the genes used to build these networks were expressed in both tissues (Additional file 3: Table S1), we can be confident that the difference in the connectivity between genes is due to differences in their relative expression levels. Thus we have identified tissue specific gene networks likely to represent functional differences between the tissues.

To understand the biological functions of the genes in the consensus modules, we performed Gene Ontology enrichment analysis using the GOrilla tool [45]. The full lists of GO analysis results for all the consensus modules can be found in Additional file 9: Table S5 and Additional file 10: Table S6. Several PAG modules showed significant functional enrichment in categories of “RNA-binding”, “translation”, “developmental process”, “immune response” and “synaptic transmission”. The functional enrichment for GO categories in the cortex was weaker than in the PAG showing only significant functional enrichment for categories involving “protein modification” and “signal transduction”.

**Fig. 6:**
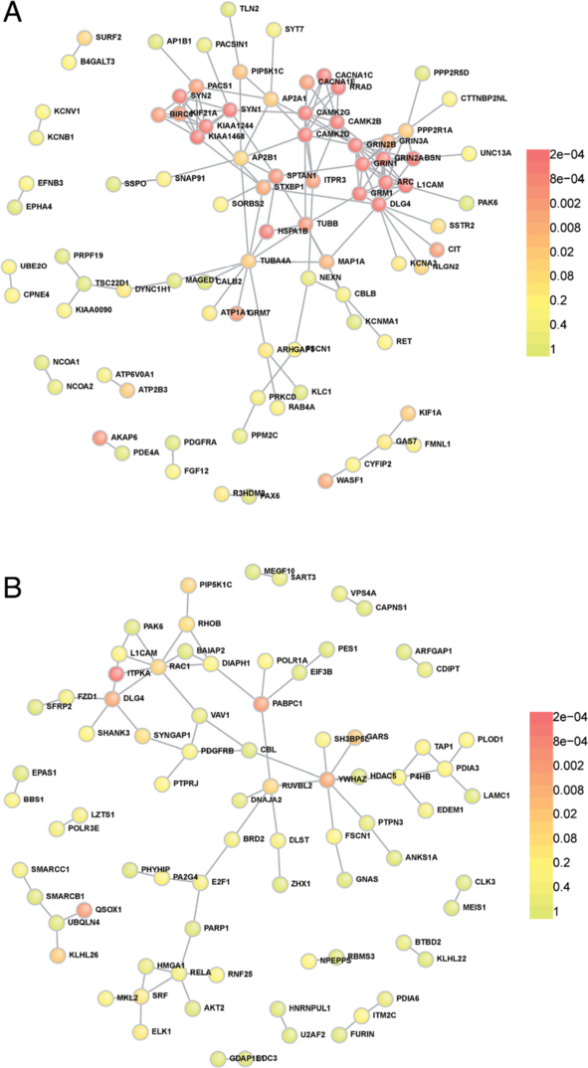
The PAG midnight blue module and cortex red module show enrichment in known protein-protein interactions. Protein-protein interactions were plotted using the DAPPLE tool. The proteins are coloured according to the significance of the degree of interactions tested with 5000 permuted networks. Genes with significantly higher degrees of direct protein interactions than expected (p-value ≤ 0.02) form a tightly clustered centre. **a** Most proteins within the midnight blue PAG module are significantly enriched with direct interactions, with the strongest connections amongst synaptic proteins such as the glutamate receptors; GRMs (mGluR) and GRINs (NMDA), highlighting their importance as hub genes. **b** Enrichment of direct interactions is overall much weaker in the cortex red module

To further test biological significance of the modules that we had uncovered, we examined the protein-protein interactions (PPI) in the most highly connected PAG (midnight blue) and cortex (‘red’) modules using DAPPLE [46]. This tool uses a within-degree permutation method with reference to the high-confidence protein-protein interaction database, InWeb, to reveal statistically significant PPIs within a given gene list. Results are illustrated in Fig. 6, where known direct interactions are shown as edges, and the significance of each protein in the module is measured by its observed number of interactions in comparison to the module size with a P value estimated through permutation. The most highly connected module in the PAG was the midnight blue module (330 genes) containing 145 direct protein interactions (Fig. 6a). Of these, the strongest interactions (p-value ≤ 2x10^−2^) involved genes in synaptic transmission, particularly amongst the glutamate receptor genes, GRINs (glutamate receptor, ionotropic NMDA) and GRMs (glutamate receptor, metabotropic), calcium/calmodulin-dependent protein kinases (CAMKs) and DLG4 (also known as PSD-95), all of which are known to localise to the post-synaptic density of glutamatergic synapses to control synapse activity and plasticity [47]. In contrast the cortex ‘red’ module (239 genes) contained 77 direct interactions (Fig. 6b). However the interactions were much more dispersed compared to the PAG midnight blue module, with fewer genes having significantly more interactions than expected (p-value ≤ 0.02). This indicated that the PAG midnight blue module was more functionally enriched than the cortex ‘red’ module and as such we focused our investigation on the functional relevance of this network of genes.

### A highly connected module related to excitatory synaptic transmission

**Fig. 7:**
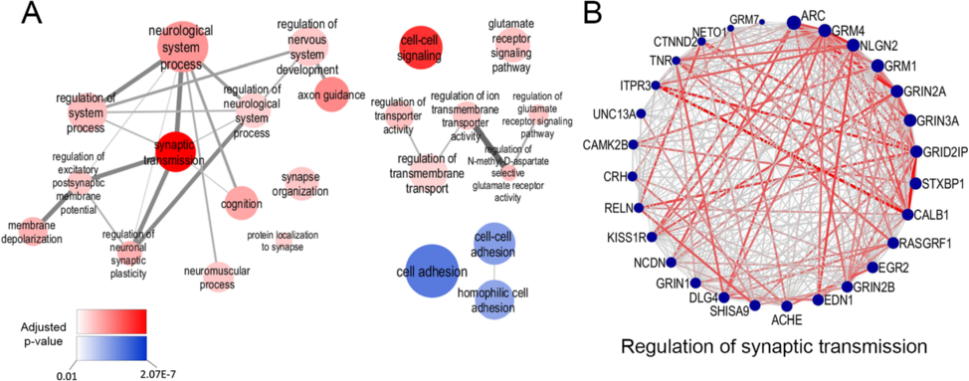
The midnight blue PAG module represents a highly connected module of genes involved in synaptic functions. **a** Gene Ontology of the midnight blue PAG module. Highly significant enrichment of gene ontology categories were observed in the midnight blue PAG module including GO terms related to cognition, nervous system development, synaptic function, glutamatergic synaptic transmission and cell adhesion. Categories linked to synapse function are shaded red and categories linked to cell adhesion are shaded blue. The level of significance for each category determines the intensity of shading, as per inset key. **b** Connectivity map of all genes involved in synaptic transmission within the midnight blue module. The topological overlap (TO) is indicated by the thickness and colour of the lines, with red lines representing the closest overlap/strongest connectivity. Nodes represent the connectivity value ‘Kw’ which represents the overall connectivity within the module for that gene. Larger nodes indicate higher connectivity. ARC (an immediate early gene that acts at the synapse) and glutamatergic synaptic receptors (such as GRM1/4, NLGN2 and GRIN2A/3A) represent hubs for these synaptic networks

In addition to strong functional enrichment illustrated by protein-protein interactions, the midnight blue module was also the most highly connected and conserved module identified in the PAG. This module displayed the top percentage overlap (80.7 %, p-value <2.2e-16) using the two clustering methods (Fig. 3a), and its intra-modular connectivity (a measurement of the degree of connectivity of each gene) was also distinctly higher than for other modules (Additional file 11: Figure S5). GO enrichment analysis revealed a significant enrichment for a number of GO categories such as synaptic transmission, synapse organisation, regulation of neuronal synaptic plasticity, glutamate receptor signalling and regulation of N-Methyl-D-aspartate (NMDA) selective glutamate receptor activity (Additional file 9: Table S5 and Fig. 7a). This suggested that the midnight blue module contains a strong synaptic component and may play an important role in synapse signalling and organisation. To determine the central genes in this module, we identified the hub genes involved in a representative GO category; “Regulation of synaptic transmission” by mapping the connectivity amongst genes within this category (Fig. 7b). This highlighted glutamate receptors (GRINs and GRMs) as some of the strongest hub genes for this GO category (4 out of 6 of the most highly connected genes), suggesting a key role for glutamatergic synapse function in this module.

**Fig. 8:**
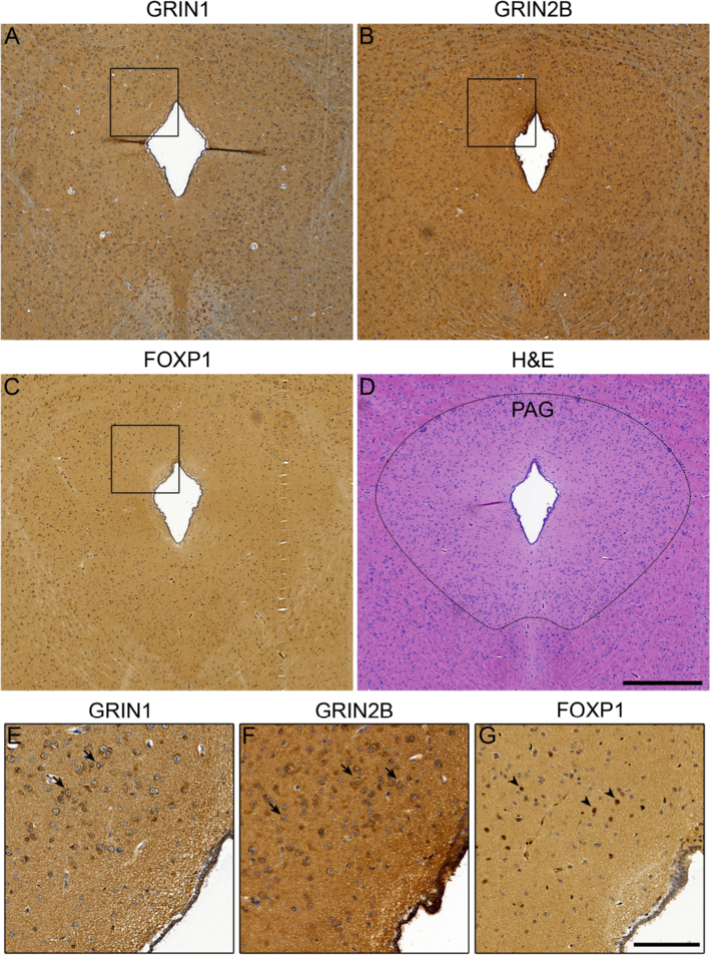
Midnight blue hub genes are expressed in the PAG of the bat brain. Protein expression pattern of (**a**) GRIN1 (**b**) GRIN2B and (**c**) FOXP1 in the bat PAG. **d** Haematoxylin and Eosin (H&E) staining of the bat PAG to show tissue structures - the boundary of the PAG has been demarcated with a dotted line. Higher magnification photos show (**e**) GRIN1 and (**f**) GRIN2B in the cytoplasm of cells within the PAG as indicated by the dark brown staining (arrow), whereas (**g**) FOXP1 could be found in nuclei (arrow head) as expected for a transcription factor. A regulatory relationship for GRIN2B and FOXP1 has previously been shown in the mouse. The overlapping expression pattern in regions of the bat PAG, suggest that in a subset of cells FOXP1 may be able to regulate *GRIN2B* expression. The scale bar represents 500 μm for a-d (d) and 125 μm for e-g (g)

In neonatal and adult tissue, NMDA receptor complexes are largely composed of GRIN1/2A or GRIN1/2B heterodimers and GRIN1/2A/2B tri-heteromeric complexes [48]. Thus, to determine if these module members were co-expressed in the same regions of the PAG we performed immunohistochemistry in *P. discolor* brain slices. Both GRIN1 (NR1 protein) and GRIN2B (NR2B protein) were highly expressed in cells throughout the PAG (Fig. 8), suggesting an overlapping expression pattern that would allow functional interactions. GRIN2A (NR2A protein) expression could not be confirmed as there are no antibodies available that recognise the sequence of the *P. discolor* NR2A protein.

Given the predicted functional relationships between genes in our network, we hypothesised that some network members may represent transcription factors and their regulatory targets. A number of neurodevelopmentally important transcriptional regulators were present in the midnight blue module, including *PAX6*, *FOXP1* and *CHD8. FOXP1* has been previously implicated in speech and language delay and disorders with communicative elements such as intellectual disability (ID) and autism spectrum disorder (ASD) [49, 50], prompting us to look for FOXP1-target relationships within the midnight blue network. In this manner, we identified 22 genes in the midnight blue module, including *GRIN2B,* that had been identified as *FOXP1* targets in at least two independent datasets (Additional file 12: Table S7) [51–53]. In the PAG, *FOXP1* was expressed in several regions and coincided with the NR2B expression pattern (Fig. 8), suggesting that in overlapping areas, FOXP1 may regulate *GRIN2B* expression in the bat brain.

In summary, the high number of protein-protein interactions, together with the overlapping expression patterns and the presence of validated transcription factor-target relationships in the midnight blue module, underscores the likely biological relevance of the network, suggesting that we have identified a functional network of genes that act at the synapse of PAG neurons.

## Discussion

This study represents the first molecular interrogations of the *P. discolor* bat; a highly promising model system for the study of vocal communication. Bats have not classically been used as genetic model organisms, partly due to difficulty in maintaining laboratory colonies and a lack of available genomic information/tools. *P. discolor* thrives in captivity and has become a well-established model for psychoacoustic research due to their highly social colony structure and complex innate and learned vocalisations [7, 14]. To address the fundamental gap in genomic understanding of this organism we have performed the first transcriptomic analysis in this species and developed a novel methodology that we used to identify biologically relevant gene networks in a conserved mammalian vocalisation brain region. Together this work establishes *P. discolor* as a novel neuro-molecular model for the study of vocal communication in mammals.

To identify functional, biologically relevant gene networks, we utilized two contrasting co-expression network analysis methods with distinct underlying algorithms; WGCNA and MCLUST. These algorithms have been shown to outperform other similar methods [28] and have individually been successfully applied to identify functionally related gene networks [17, 19, 23, 28]. However when using the standard settings, neither WGCNA nor MCLUST could generate stable gene networks with our small sample size (N = 6 per condition). This was attributed to the large number of genes showing substantial uncertainty in cluster assignment and could thus be randomly assigned to multiple clusters during analysis. The recommended sample size for performing WGCNA is 15-20 samples per condition (http://labs.genetics.ucla.edu/horvath/CoexpressionNetwork/Rpackages/WGCNA/faq.html), and for MCLUST there is no clear documentation on the optimal sample size. However, it was clear from our analysis that neither algorithm could successfully generate stable gene clusters with 6 samples.

Co-expression network algorithms such as WGCNA retain highly uncertain genes within the dataset typically assigning them to what is termed an “unclusterable group”, sometimes called the “grey module”. However, in our study we have shown that removing these borderline genes prior to re-clustering remaining genes can result in radical changes (Additional file 5: Figure S3). This is a notable finding, as it indicates that uncertain genes can cause disturbances to the final networks if retained, even when assigned as “unclusterable”. Including these genes while clustering is likely to affect the distribution of gene expression features resulting in a more dispersed, less stable cluster. To overcome this limitation and improve network stability, we devised a method to sequentially remove these borderline genes using iterative re-clustering. This resulted in greatly improved cluster stability for both WGCNA and MCLUST with our sample size, producing smaller but more robust clusters highly likely to represent functional gene networks (see below). Given how the iterative procedure improved network stability we suggest that it will also result in improved network building when large sample sizes are available. Thus it will be valuable to re-analyse published co-expression gene network datasets as including iterative re-clustering may strengthen biological findings and/or yield potentially novel insights.

To further refine the gene networks obtained following iterative re-clustering, we compared the networks identified by WGCNA with those identified by MCLUST to produce consensus modules. WGCNA and MCLUST employ very different algorithms, thus the consensus modules represent robust gene networks resistant to methodological differences. We found extensive and significant overlap using these contrasting clustering methods in both PAG and cortex. In contrast, little overlap was observed when comparing the consensus modules of the two tissues with each other, indicating that the gene networks identified represent tissue specific gene networks. In conclusion, we show here that using our combinatorial iterative re-clustering approach, high confidence, tissue specific functional gene networks can be built with a limited number of samples. This is significant, as obtaining large numbers of samples can be prohibitive for investigating co-expression gene networks in clinical or post-mortem tissue, or non-model animals where sample size is limited. With our approach we have overcome this limitation, opening up new avenues of investigation for such studies.

Using our novel methodology we identified strongly connected functional gene networks in both the PAG and cortex of *P. discolor*. The PAG midnight blue module was the most strongly connected and conserved module identified, and gene ontology analysis showed enrichment for genes involved in synaptic development and function. Specifically, genes involved in glutamate receptor signalling and regulation of NMDA selective glutamate receptor activity were the most connected hub genes within the module, suggesting that these pathways are important for PAG function. Furthermore the high number of known protein-protein interactions in the midnight blue module provided strong evidence that our method produced functional gene networks. When we explored the known roles of the genes within the midnight blue module the functional connectivity of these genes was even more striking (see Fig. 6a). A number of NMDA receptor (NMDAR) subunits were hub genes within the network, including subunits that compose the predominant heteromeric complexes in the postnatal brain (GRIN1/2A/2B). Proteins that interact with NMDA receptors at the synapse such as DLG4 and CTRO (CIT gene) formed significantly connected networks with the NMDAR subunits in the midnight blue module. DLG4 (PSD-95) also serves as a bridge to other synaptic proteins significantly connected in the midnight blue network including MAP1A, CAM kinases (CAMK2B, CAMK2D and CAMK2G) and neuroligins (NLGN2) [54]. Signalling through NMDA receptors can activate the MAPK signalling pathway leading to transcriptional changes [54], with one of the most robustly activated genes being the immediate early gene activity-regulated cytoskeletal-associated protein (ARC) [55]. ARC was also found in the midnight blue module and was one of the strongest hub genes, showing significant links with the co-expressed NMDA receptor subunits in the PAG. Taken together, this convergence of connected proteins at the post-synaptic density of glutamatergic synapses suggests that we have uncovered a biologically relevant network acting at the post-synaptic density in the mammalian PAG.

Our evidence indicates that we have identified a biologically relevant gene network, but what functions are mediated by this post-synaptic pathway? The PAG has been implicated in key neuronal processes; pain and analgesia, fear and anxiety, lordosis, cardiovascular control and vocalisation [56]. Indeed, NMDA receptors have been implicated in the regulation of some of these processes in the PAG [57]. However the precise role remains unclear as NMDA has been found to enhance, inhibit or have no effect on analgesia and cardiovascular control depending on the study in question. By far the most consistent role of NDMA signalling in the PAG is its role in promoting vocalisation. For example, comprehensive pharmacological studies have shown that injection of glutamate agonists, especially NMDA receptor agonists, in the PAG of squirrel monkeys were highly effective at inducing vocalisations [41]. Similarly, this effect could be reproduced in sedated cats [42], and in our bat model (*P. discolor*) activation of kainate glutamate receptors could reliably elicit vocalisations from the PAG [39]. In contrast, activation or inhibition of other synaptic transmission receptors in the PAG such as glycine, noradrenaline, dopamine and serotonin receptors did not result in vocalisation suggesting that glutamatergic, but not general synaptic activation in the PAG, is required for promoting vocalisations [41]. Importantly, glutamate receptors have not only been shown to be sufficient, but also necessary for PAG mediated vocalisations, as vocalisation signals initiated from the anterior cingulate cortex, a brain region upstream of the PAG in the vocal initiation pathway, can be blocked by injection of either glutamate or NMDA receptor antagonists into the PAG [58]. Thus, the consistent and established role of NMDA for promoting vocalisations from the PAG together with the strong overrepresentation of NMDA pathway genes in the midnight blue network suggests that this gene network is likely to be mechanistically important for the evolutionarily conserved function of the PAG in mammalian vocalisation.

In support of the role of the midnight blue network in vocalisation, a number of module members have been linked to vocalisation phenotypes in other species. Expression levels of *Grin* receptors are linked to pro-social ultrasonic vocalisations in rats [59], and in humans *GRIN2A* mutations are associated with idiopathic focal epilepsy (IFE) alongside speech defects such as verbal dyspraxia and dysphasia [60, 61]. *FOXP1* has also been found to cause speech and language delay, ID and ASD in human patients [49, 50]. *P. discolor* provides an ideal model to further investigate the role of these and other midnight blue network members in vocalisation related phenotypes as genetic tools have already been developed that will allow us to manipulate gene expression changes [62] and observe corresponding effects on features of complex vocalisations in these bats. Vocal production pathways are highly conserved among mammals [33], and as such, interrogations in bats will be highly relevant for understanding these pathways in other mammalian species, including humans. In the future, extending our approach to other brain regions involved in vocalisation and combining this with behavioural assays (e.g. of vocal learning) will not only give insight into the molecular mechanisms required for a functioning vocal communication system, but may also shed light on the evolution and development of spoken language [16].

## Conclusions

We have developed an innovative approach to cluster co-expressing gene networks and shown that the method is highly effective in detecting robust functional gene networks with limited sample sizes. This method represents a significant advantage over standard network analysis that will be of particular use when studying rare disorders or where samples are limited, such as in this study. Using this approach we identified a highly inter-connected module centred on glutamatergic synaptic transmission that is likely to be associated with the vocalisation function of the PAG. This represents the first molecular interrogation of *P. discolor* and establishes this bat as an exciting new model system for understanding the genetics of mammalian vocal communication.

## Methods

### Animal housing and conditions

All bats (*P. discolor*) originated from a breeding colony in the Department Biology II of the Ludwig-Maximilians-University in Munich. In this colony animals were kept under semi-natural conditions (12 h day / 12 h night cycle, 65 to 70 % relative humidity, 28 °C) with free access to food and water. All experiments complied with the principles of laboratory animal care and the regulations of the current version of the German Law on Animal Protection. Animals were euthanized by an intraperitoneally applied lethal dose of pentobarbital (0.16 mg/g bodyweight). According to German Law on Animal Protection a special ethical approval is not needed for this procedure, but the number of sacrificed animals was reported to the district veterinary office.

### Brain dissection and tissue punching

Animals were euthanized by an intraperitoneal lethal dose of pentobarbital (0.16 mg/g bodyweight) and decapitated prior to brain removal. Subsequently, the brains were transferred to ice cold dissection buffer (saccharose 210 mM, KCl 3 mM, MgCl_2_.6H_2_O 3 mM, NaHCO_3_ 23 mM, NaH_2_PO_4_.H_2_O 1.2 mM, glucose 11 mM in DEPC treated water). Brains were then embedded in 3 % low melting point agarose (Sigma) dissolved in DEPC treated water and sliced into 500 μm thick sections using a VF-200 vibratome (Precision instruments). The PAG was dissected using either 1 mm or 2 mm tissue punches (Miltex) depending on the size of the PAG per section (Additional file 1: Figure S1). Corresponding cortical punches were taken from every brain slice where the PAG was dissected (Additional file 1: Figure S1). Tissue punches for corresponding brain regions were pooled, transferred to RNA later solution (Qiagen) and frozen for subsequent RNA extraction.

### RNA extraction and purification

Tissue punches were thawed at room temperature and lysed using TRIzol (Life technologies) reagent. Briefly, tissue punches were homogenized in 500 μl of TRIzol using a 21G syringe needle and incubated for 15 min at room temperature. 100 μl chloroform was added and mixed by shaking. The samples were then centrifuged at 12,000 g for 10 min at 4 °C. The aqueous layer containing the RNA was diluted 1:1 with 70 % ethanol and purified using the RNeasy micro kit (Qiagen) as per the supplier’s instructions including the optional on column DNase digestion step. Eluted RNA was analysed for RNA integrity using Agilent RNA 6000 Nano Kit (Agilent technologies) following the suppliers instructions. Samples meeting the quality criteria (≥200 ng total RNA, RIN ≥ 7 and 28S/18S ≥ 1) were shipped to the Beijing Genomics Institute (BGI) in dry ice for RNA-sequencing.

### Transcriptome assembly and annotation

*De novo* assembly of the *P. discolor* transcriptome was done using the Trinity package [63] under default settings. After Illumina paired-end RNA sequencing, cleaned fastq sequences with minimum base quality 20 were used in the assembly. Coding sequences were extracted using transdecoder (within the Trinity package). The predicted open reading frames were then compared to known proteins from Uniprot database by BLAST+ with E-value cutoff 0.001. In many cases multiple alternatively spliced transcripts could be matched to a single protein, thus identified as isoforms.

To estimate abundance of each transcript, the paired-end reads of each bat sample were mapped to the assembled transcripts using RSEM [64] within Trinity. The abundance of isoform transcripts were compared and the major isoform of a gene was defined as the transcript to which the greatest percentage of sequence reads were mapped. We used edgeR [65] to identify differentially expressed transcripts. The transcripts count was normalized by the trimmed-mean of M values (TMM) normalization method implemented with edgeR, and the 6 PAG and 6 cortex samples were treated as replicas for the two brain regions respectively during differential expression analysis. Transcripts with ≥ 2 fold change and multiple testing adjusted P-value ≤ 0.01 were identified as differentially expressed.

### Clustering analysis

We applied an iterative re-clustering procedure to identify stable gene clusters. First, the gene-to-cluster identity could be determined by module membership, a fuzzy measure of how closely a gene is associated with the module, in WGCNA or clustering uncertainty, which is a probabilistic measure of how unlikely the gene belongs to a certain cluster, in MCLUST. At each round of re-clustering, genes with module membership (WGCNA) < 0.8 or uncertainty (MCLUST) > 0.01 were removed. The new modules were compared to the previous modules pair-wisely to find the best matching pairs. The percentage of genes in the new module that were also found in the previous module was used to assess the quality of module preservation. The average of all the module preservation values was used to assess the stability of clustering. With the removal of outliers at each round of re-clustering, the stability of gene modules increased.

The iterative re-clustering was done for WGCNA and MCLUST clustering in parallel. WGCNA was utilized according to Hilliard et al. [19] with the R library WGCNA. During each round of re-clustering, the soft power was re-determined as the first point where the R^2 value approached 0.9. The resulting soft powers for all rounds of re-clustering were between 12 and 18. The minimum size of the modules were set at 100 and cut height at 0.2. The MCLUST method [23] was used according to the MCLUST R package user manual.

### Module overlap analysis

The overlapping of modules between PAG and Cortex and between WGCNA- and MCLUST- produced modules was determined by all-against-all pairwise comparisons with a custom perl script. Thus each module from one tissue/method has one best matching counterpart from the other. The percentage of overlap shown in the Figs. 3 and 4 was calculated as (Number of overlapping genes)/(Number of genes in the smaller of the pairwise modules)x100. The overlapping significance was calculated using Fisher’s exact test.

### Gene ontology (GO) and protein interaction network analysis

GO enrichment analysis was performed using the GOrilla tool. The testing gene lists were genes in the conserved modules, and the background list was the expressed major isoforms in PAG or cortex. The enriched GO terms were then collapsed based on similarity (set at medium threshold, 0.7) using REVIGO. The GO term plot was generated using Cytoscape (version 3.0.2). Protein interaction network analysis was done using DAPPLE (http://www.broadinstitute.org/mpg/dapple/dapple.php) with the default settings and 5000 permutations.

### Immunohistochemistry

*P. discolor* whole brains were fixed in 10 % formalin solution for 24-48 hours. Fixed brains where sliced coronally, paraffin embedded and sliced into 4 μm thick sections. For staining, sections were de-waxed in Xylene (Sigma) for 10 min and washed four times in 100 % ethanol, once in water and once in PBS. Antigen retrieval was done in either pH6 citrate buffer (Immunologic) (for GRIN1 and GRIN2B) or pH9 Tris-EDTA buffer (Immunologic) (for FOXP1) in a microwave for 3-5 min at 850 Watts and 10 min at 180 Watts. Endogenous peroxidase was blocked for 10 min with 3 % H_2_O_2_ (Sigma) diluted in methanol. Sections were washed in water and blocked in 10 % normal goat serum (Vector labs) for at least 30 min at room temperature. The tissue was then encircled with a PAP pen and stained overnight at 4 °C with either 1:100 anti-rabbit NR1 (GRIN1) (MAB363 Millipore), 1:50 anti-rabbit NR2B (GRIN2B) (06-600 Millipore) and 1:10 anti-rabbit FOXP1 (ab134054 Abcam) diluted in 10 % normal goat serum. Sections were washed three times in PBS and secondary incubation was done with 1:1000 goat-anti-rabbit HRP (Vector labs). Sections were then washed three times in PBS followed by incubation with avidin-biotin-horseradish peroxidase complex (ABC) using the Vectastain kit (Vector Laboratories). Briefly, reagent A and reagent B were diluted together 1:100 in PBS and incubated for 30 min at room temperature prior to addition to the tissue sections. Tissue sections were then incubated for 45 min at room temperature. Sections were washed again in PBS three times before adding DAB (Immunologic). The color reaction was allowed to develop for 7 min. Sections were then washed in water once and counterstained for 1 min with Haematoxylin modified (Harris and Gill II) (Sigma). After removal of excess counterstain, sections were washed four times in 100 % ethanol and twice in Xylene (Sigma). Finally, sections were coverslipped using DPX (Sigma).

## Description of supporting data

The data sets supporting the results of this article are included within the article (and its additional files). The raw reads from the RNA sequencing have been deposited in the NCBI bioproject repository (Accession: PRJNA291690 and ID: 291690) and can be found at http://www.ncbi.nlm.nih.gov/bioproject/291690.

## Additional files

Additional file 1: Figure S1. PAG and cortex sample collection from fresh brain. Photos of fresh brain slices (A) before and (B) after sample collection with tissue punches. The PAG is shown by an arrow. The cortical punches were always taken from the same region illustrated in (B). The scale bar represents 5 mm respectively for (A) and (B). (JPEG 268 kb) Additional file 2: Figure S2. Genes differentially expressed between the cortex and PAG. Unsupervised hierarchical clustering of the major isoform for each expressed gene demonstrated differentially expressed genes between the PAG and cortex. The colour key indicates log_2_ fold change. The PAG and cortex samples are clearly separated into 2 groups by hierarchical clustering under the default setting (distance = Euclidean, method = complete). (JPEG 405 kb) Additional file 3: Table S1. Cortex and PAG expression differences FDR 0.001 (XLSX 1031 kb) Additional file 4: Table S2. Gene ontology of top 500 overexpressed genes for cortex and PAG (XLSX 50 kb) Additional file 5: Figure S3. Module preservation during iterative re-clustering of modules. The gene to module assignment was initially unstable due to genes with a high uncertainty being present in the dataset. During iterative re-clustering module preservation was calculated as the percentage of genes of the previous clusters retained in the best-matching new clusters. An iterative clustering procedure was applied to both WGCNA and MCLUST by first removing uncertain genes and then re-clustering with the same parameters. The average module preservation increased during iteration, and reached a maximum, where additional iterations did not improve average module preservation. (JPEG 1039 kb) Additional file 6: Figure S4. Iterative re-clustering of WGCNA and MCLUST modules. Genes with an uncertainty value greater than 0.01 in MCLUST or a module membership value greater than 0.8 in WGCNA were gradually filtered out from the dataset to improve cluster stability. (JPEG 2265 kb) Additional file 7: Table S3. PAG ‘consensus’ module gene lists/details (XLSX 37 kb) Additional file 8: Table S4. Cortex ‘consensus’ module gene lists/details (XLSX 31 kb) Additional file 9: Table S5. Full GO for PAG modules (XLSX 83 kb) Additional file 10: Table S6. Full GO for cortex modules (XLSX 32 kb) Additional file 11: Figure S5. Intramodular connectivity of PAG modules. Boxplot shows the values of intramodular connectivities (kWithin) of all PAG modules. The intramodular connectivity (kWithin) is a measurement of the degrees of connectivity of each gene within a module. The tightness of each module can therefore be represented by the distribution of kWithin. The kWithin parameter was calculated using WGCNA. The boxplot shows the midnight blue PAG consensus module has clearly the highest average and overall kWithin among all the modules. (JPEG 267 kb) Additional file 12: Table S7. FOXP1-target relationships identified in the midnight blue network. (XLSX 23 kb)

## Abbreviations

ASD: Autism spectrum disorder
ARC: Activity-regulated cytoskeletal-associated protein
CAMK: Calcium/calmodulin-dependent protein kinases
DLG4: Disks large 4
FDR: False discovery rate
GO: Gene ontology
GRIN: Glutamate receptor, ionotropic NMDA
GRM: Glutamate receptor, metabotropic
ID: Intellectual disability
MCLUST: Model-based clustering
MRI: Magnetic Resonance Imaging
NMDA: N-Methyl-D-aspartate receptor
PAG: Periaqueductal gray
*P.discolor*: *Phyllostomus discolor*
WGCNA: Weighted gene co-expression analysis
