# Supplementary figures and images for: A novel approach identifies the first transcriptome networks in bats: a new genetic model for vocal communication
Source: BMC Genomics. 2015 Oct 22;16:836. doi: 10.1186/s12864-015-2068-1 (PMC4618519; doi:10.1186/s12864-015-2068-1)

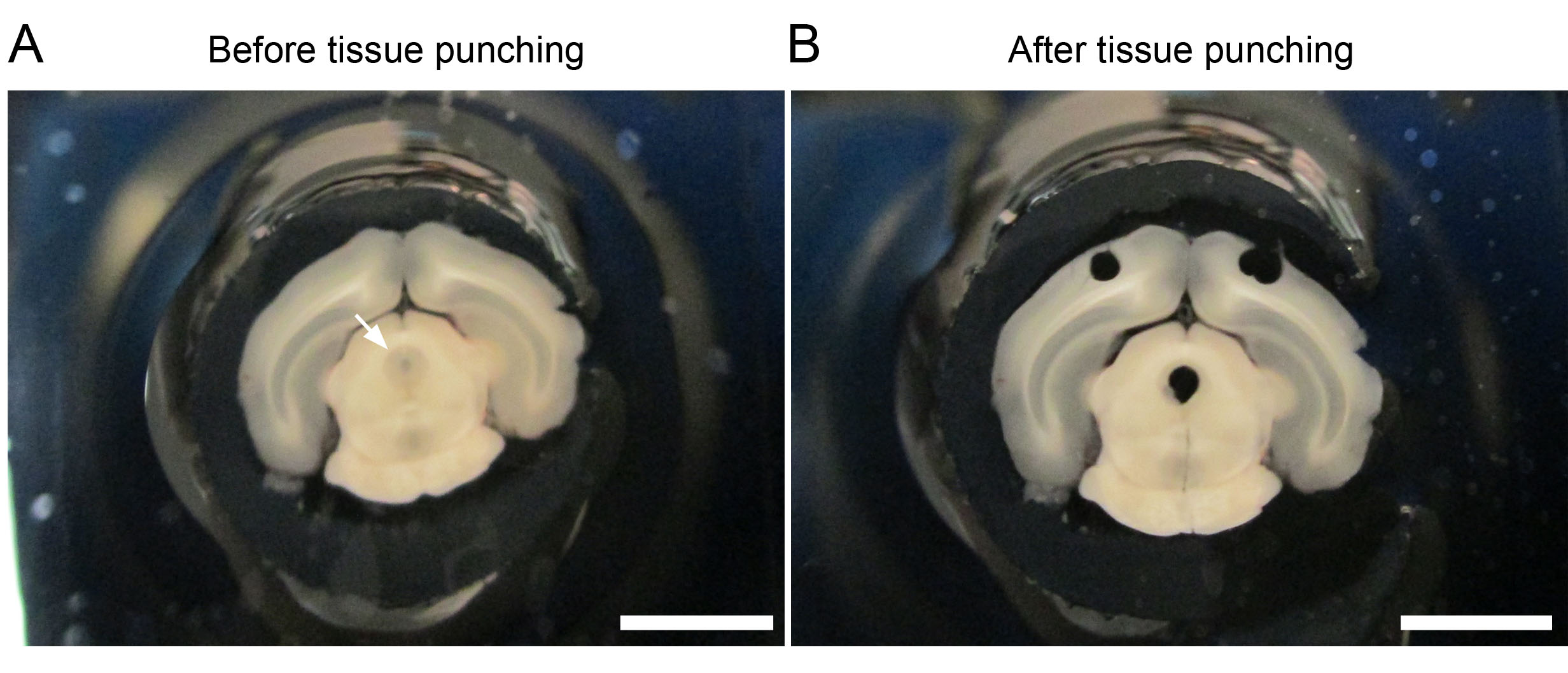

Supplement: Additional file 1: Figure S1. — PAG and cortex sample collection from fresh brain. Photos of fresh brain slices (A) before and (B) after sample collection with tissue punches. The PAG is shown by an arrow. The cortical punches were always taken from the same region illustrated in (B). The scale bar represents 5 mm respectively for (A) and (B). (JPEG 268 kb) [file 12864_2015_2068_MOESM1_ESM.jpg]

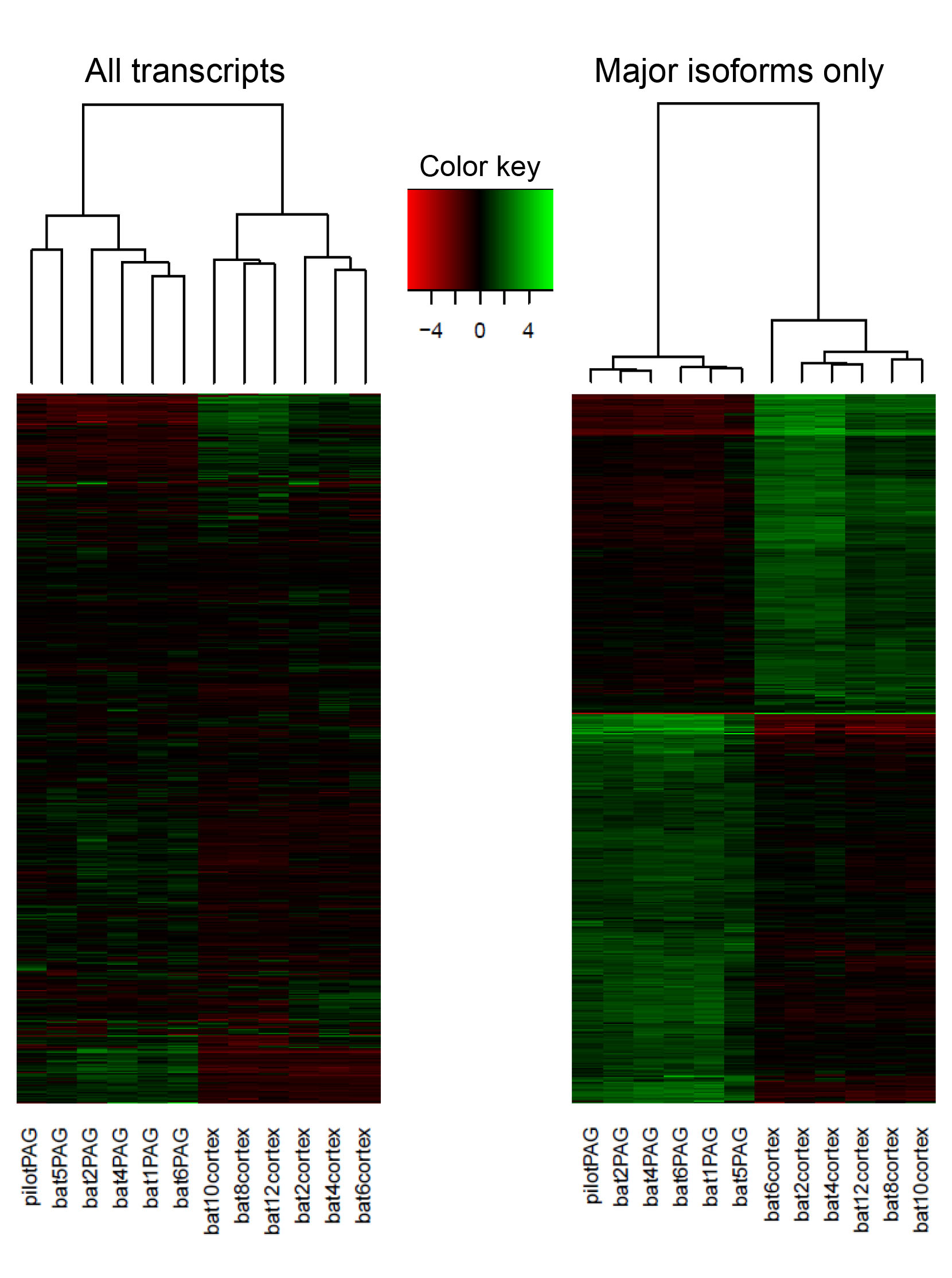

Supplement: Additional file 2: Figure S2. — Genes differentially expressed between the cortex and PAG. Unsupervised hierarchical clustering of the major isoform for each expressed gene demonstrated differentially expressed genes between the PAG and cortex. The colour key indicates log2 fold change. The PAG and cortex samples are clearly separated into 2 groups by hierarchical clustering under the default setting (distance = Euclidean, method = complete). (JPEG 405 kb) [file 12864_2015_2068_MOESM2_ESM.jpg]

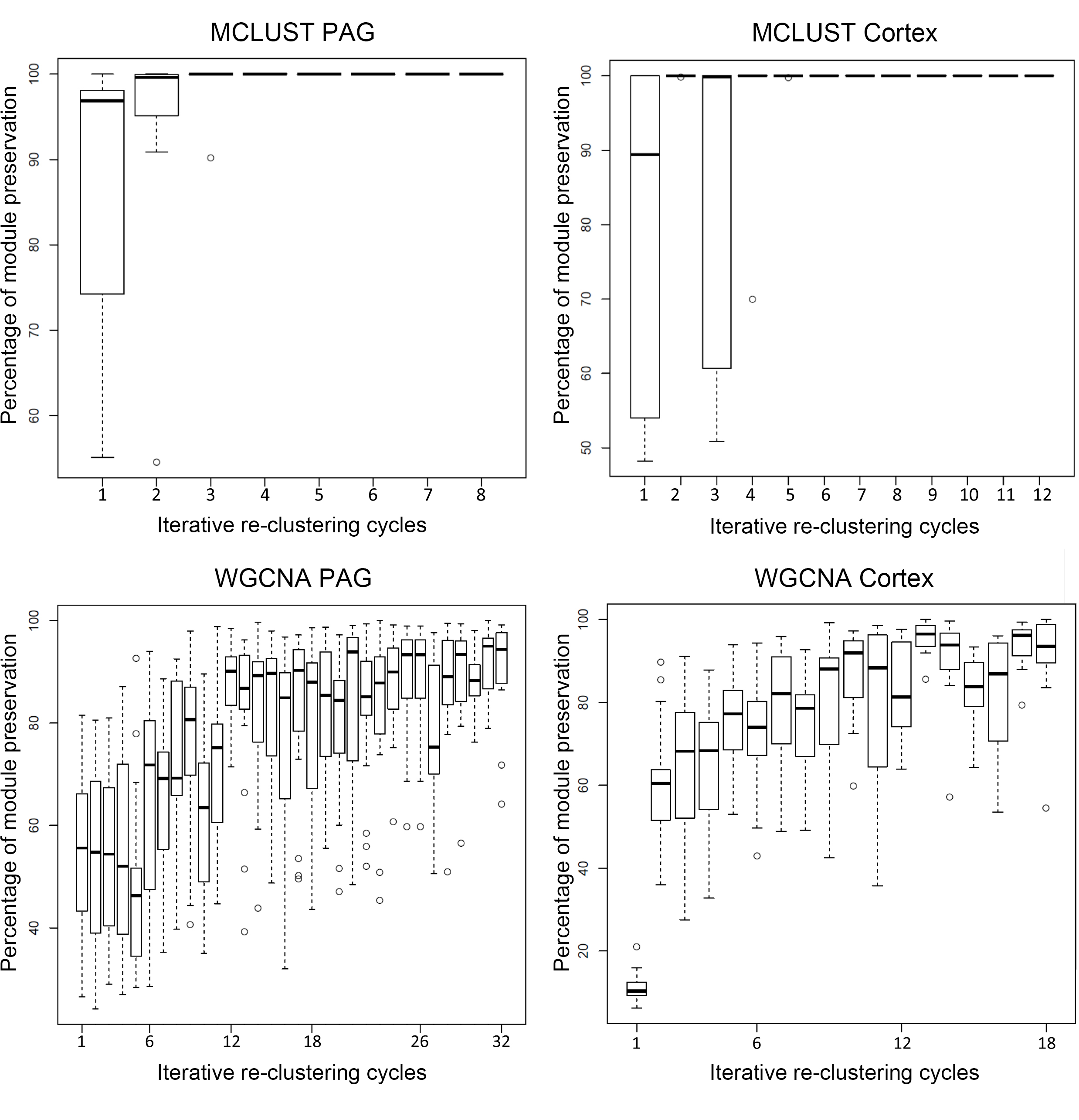

Supplement: Additional file 5: Figure S3. — Module preservation during iterative re-clustering of modules. The gene to module assignment was initially unstable due to genes with a high uncertainty being present in the dataset. During iterative re-clustering module preservation was calculated as the percentage of genes of the previous clusters retained in the best-matching new clusters. An iterative clustering procedure was applied to both WGCNA and MCLUST by first removing uncertain genes and then re-clustering with the same parameters. The average module preservation increased during iteration, and reached a maximum, where additional iterations did not improve average module preservation. (JPEG 1039 kb) [file 12864_2015_2068_MOESM5_ESM.jpg]

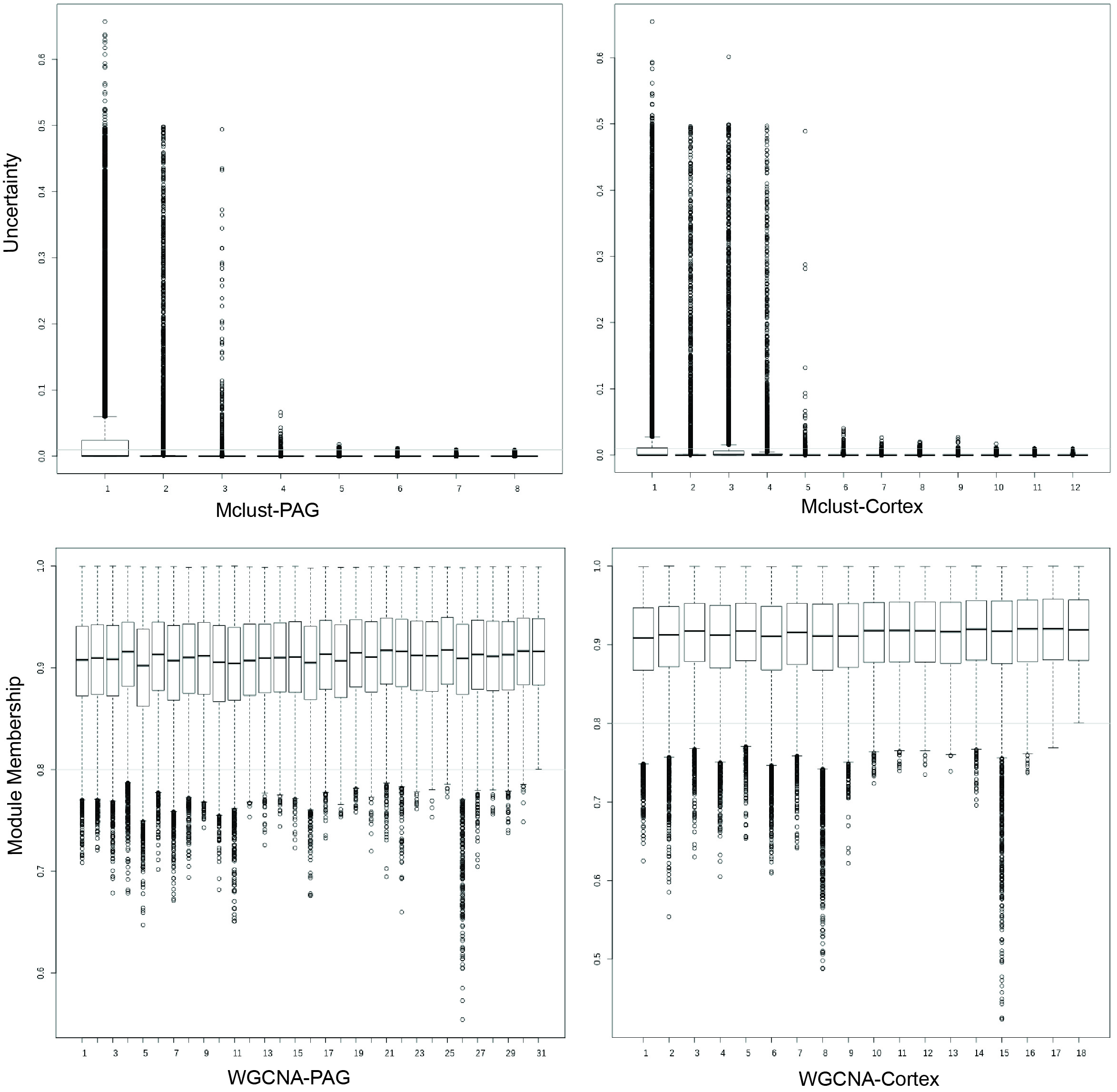

Supplement: Additional file 6: Figure S4. — Iterative re-clustering of WGCNA and MCLUST modules. Genes with an uncertainty value greater than 0.01 in MCLUST or a module membership value greater than 0.8 in WGCNA were gradually filtered out from the dataset to improve cluster stability. (JPEG 2265 kb) [file 12864_2015_2068_MOESM6_ESM.jpg]

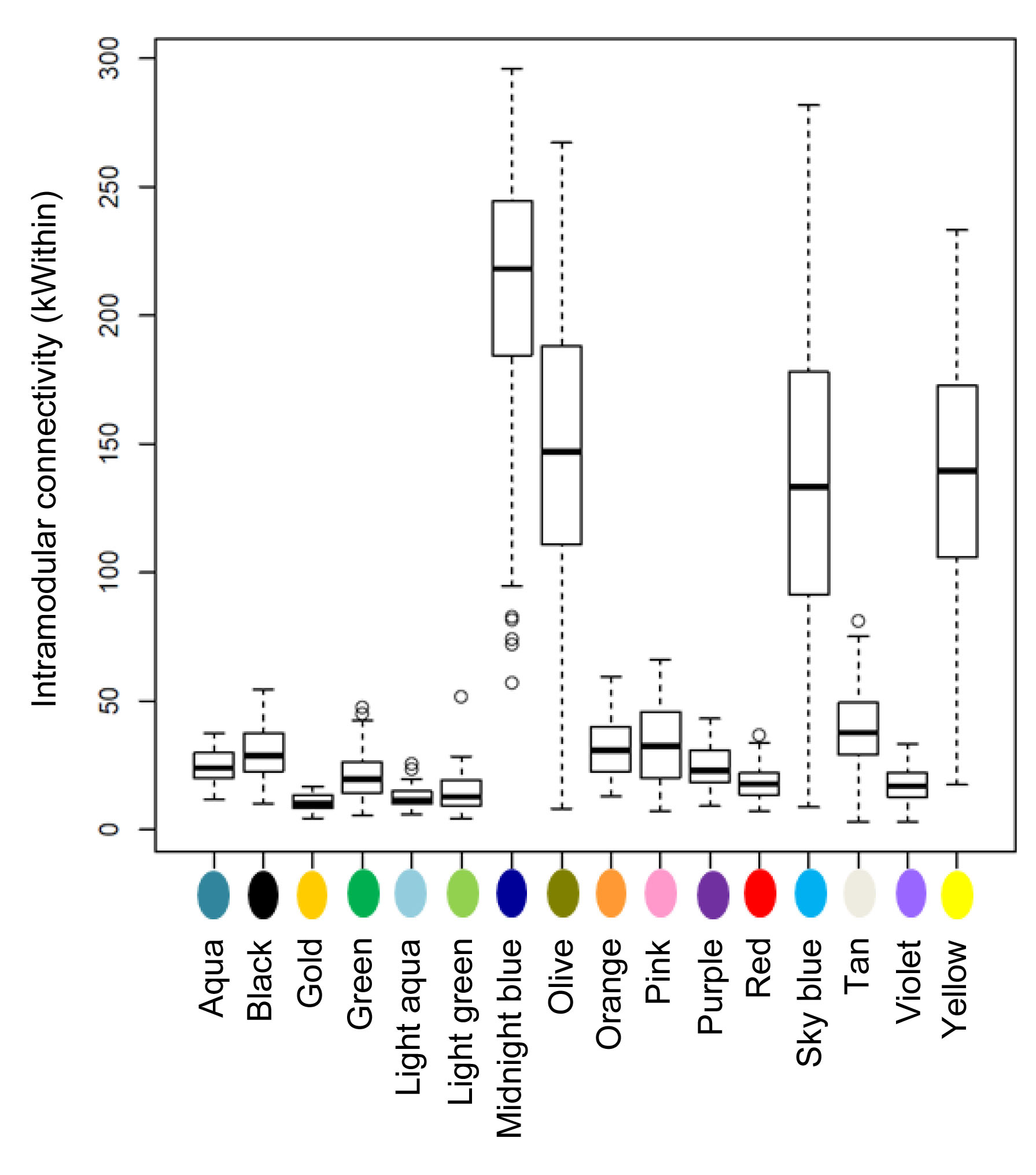

Supplement: Additional file 11: Figure S5. — Intramodular connectivity of PAG modules. Boxplot shows the values of intramodular connectivities (kWithin) of all PAG modules. The intramodular connectivity (kWithin) is a measurement of the degrees of connectivity of each gene within a module. The tightness of each module can therefore be represented by the distribution of kWithin. The kWithin parameter was calculated using WGCNA. The boxplot shows the midnight blue PAG consensus module has clearly the highest average and overall kWithin among all the modules. (JPEG 267 kb) [file 12864_2015_2068_MOESM11_ESM.jpg]
